# Cerebral localization of higher functions: The period between Thomas Willis and Paul Broca

**DOI:** 10.1590/1980-57642018dn13-020014

**Published:** 2019

**Authors:** Eliasz Engelhardt

**Affiliations:** 1 Cognitive and Behavioral Neurology Unit, INDC - CDA-IPUB - UFRJ Rio de Janeiro-RJ-Brazil

**Keywords:** higher nervous function, Willis, Broca, aphemia, speech, language, hemispheric asymmetry, função nervosa superior, Willis, Broca, afemia, fala, linguagem, assimetria hemisférica

## Abstract

The age-old debates about the localization of the mind (higher functions) took a new course when Willis located a higher nervous function (memory) in the brain parenchyma, and supposedly, in the cerebral cortex. About two centuries later, Broca, founded on solid scientific reasoning, localized a circumscribed area of the 3^rd^ frontal circumvolution of the left hemisphere as the seat of articulate language, a higher function (speech - language domain). He (and Dax) also defined the functional asymmetry (specialization) of the hemispheres, with left dominance (for language). The period between the findings of these individuals was not quiescent, as numerous authors contributed with their theoretical and clinicopathological research toward creating a conducive scientific atmosphere for this accomplishment, and should be regarded as important. Further studies, in the decades that followed, revealed the localization of additional aspects of language and of other higher functions (cognitive domains).

## RESUMO.

Os debates milenares sobre a localização da mente (funções superiores) chegaram a um novo caminho quando Willis localizou uma função nervosa superior (memória) no parênquima cerebral, e supostamente, no córtex cerebral. Cerca de dois séculos depois, Broca, baseado em sólido pensamento científico, localizou uma área circunscrita da 3ª circunvolução frontal do hemisfério esquerdo como o sítio da linguagem articulada, uma função superior (fala - domínio da linguagem). Ele (e Dax) também definiu a assimetria funcional (especialização) dos hemisférios, com dominância esquerda (para linguagem). O período entre os achados dessas duas personalidades não ficou quiescente, considerando que numerosos autores contribuíram, com suas pesquisas teóricas e clinicopatológicas, para criar uma atmosfera científica adequada para tal realização, devendo ser vistos como importantes. Mais estudos, nas décadas seguintes, revelaram a localização de aspectos adicionais da linguagem e de outras funções superiores (domínios cognitivos).

The localization of the mind (higher nervous functions, faculties) has been a focus of discussion since ancient times. After millennia of religious, philosophical, and scientific thinking, studies and speculations, the brain finally predominated over the heart as the seat of control. Following a hypothetical ventricular localization concept, a view that endured for more than one millennium, the faculties acquired a place in the brain parenchyma.^1^^,^ 2 Further studies revealed new and more precise definitions and locations for these functions, a quest that endured for several centuries.

Here, the period spanning about two centuries between Willis’ speculative proposal and Broca’s modern scientific reasoning and methods of study, will be analysed.

## Willis and Broca

Willis and Broca are seen by most authors as the first to propose, each in his own way, the localization of a higher function in the brain (Box 1).

**Box 1:** Willis and Broca, findings and excerpts.

Author	Function and localization
**Thomas Willis** (1621-1675) English physician and anatomist	Willis grounded his theories on his anatomical studies and clinicopathological cases^3^^,^ 4. He explained: ‘…some functions belong to its substance [of the brain]…amongst these…imagination, memory and appetite...”. Then: ‘…the ‘memory act’ *(memmoriae actus)* consists of regurgitation of the spirit from the outer surface of the brain [cerebral cortex]…” . He reinforced the cortical localization of memory with the manner of its acquisition (for auditory information): ‘…thence this impression spreads further, and brings to the brain the item (feature)…and leaves in the cortex its image or a private mark for the memory …”, and regarding remembrance (for vision): ‘…a sensory impression, like the optical forms… agitates the cortex of the brain…the sensible objects are impressed as icons (images) or characters, and when later reflected back from thence, revive the memory of the same thing” (1664)^3^. It is noteworthy that Willis named and described several brain structures, and the arterial circle of the base, named after him; he created the term ‘neurology’ (**νευρολογία** [neurologia – in Greek) to designate the study of peripheral nerves, later extended to denote the speciality^3^.
**Pierre-Paul Broca** (1824-1880) French physician and anthropologist	Broca studied patients with speech loss (1861-1865) - his two initial cases, Leborgne (*Tan*) and Lelong, were examined in life, and their condition was named ‘aphemia” (*aphèmie*) (1861); their post-mortem examinations of the brain revealed, in both cases, a lesion to an area in the left frontal lobe. Further cases provided confirmation of this finding, and he was able to conclude: ‘…the site of production of the articulate language (speech) (*langage articulé*) was localized… in the posterior part of the 3^rd^ frontal circumvolution of the left hemisphere, and that a lesion there resulted in aphemia” ^5,6,7^. He also recognized a functional difference between the hemispheres, and for speech, he stated: ‘...we speak with the left hemisphere” (...*nous parlons avec l’hèmisphère gauche*) (1865)^6^.It should be highlighted that Broca’s study on speech may be regarded as a foundation stone for aphasiology.

First, Thomas Willis localized some functions in the brain parenchyma, such as imagination, memory and appetite,^3^^,^ 4 with the memory in the cerebral cortex, dependent on the flow of the animal spirit, and exemplified by the processes of memory acquisition and remembrance (1664).^1^^,^ 3 He is credited for the primacy to localize a higher function in the brain (cerebral cortex), although presumptively, and without further precision. However, Willis’ localization ideas gradually waned.

About two centuries later, Pierre-Paul Broca reported his extraordinary finding, a result of scientific thinking and a stroke of luck. He examined patients with speech loss, some followed by pathological verification, and concluded that the production of articulate language (speech) was related to the posterior part of the 3^rd^ frontal circumvolution of the left hemisphere, where a lesion at this site resulted in ‘aphemia’ (1861-1865).^5^^-^ 7 Thus, Broca demonstrated, for the first time, a circumscribed specific area related to a higher function, namely, language (speech).

Broca also established that the two hemispheres were different (functional asymmetry, lateralization, specialization) and affirmed the dominance of the left side for speech (1865).^7^ The primacy of the hemispheric asymmetry proposition constituted a matter of dispute with Gustave Dax, representing his father, the late Marc Dax (see also below).^8^

Thus, the establishment of a scientifically grounded ‘localization of a higher function’ and the concept of ‘hemispheric specialization’ stemmed from the study of a language disorder.

## After Willis and before Broca

The period between these findings was not a quiescent one, being marked by works of numerous authors. Their studies were based on an almost purely theoretical approach (Swedenborg, Prochaska, Gall) or on clinicopathological analysis of brain lesion cases (Wepfer, Valsalva, Pourfour du Petit, Morgagni, Lallemand, Andral, Rostan, Bouillaud, Dax, among others). Many of these investigations, apparently, were not specifically searching for a location in the brain of higher functions. Nevertheless, numerous reported cases cite speech disorders, but without further comments. Some authors will be highlighted for their special contribution. However, all should be seen as important and certainly helped provide the right environment for such ideas^2^ (Box 2).

**Box 2:** Theoretical-based view of localization, findings and excerpts from the authors.

Author	Function and localization
**Emanuel Swedenborg** (1638-1772) Swedish scientist and philosopher	Swedenborg considered that: ‘The [surface of the] cerebrum is made up…discrete cortical parts… designated ‘sphaerulae’ or ‘cerebellula’…related to each sense and other functions… thus permitting the cerebrum to perform at the same time, and yet distinctly, so many different tasks”^9^. Concerning the seat of the intellect [higher functions]…he affirmed: ‘…the ‘anterior province’ of the cerebrum (highest lobe)[frontal lobe] is more important than the posterior”, and explained: ‘If this portion of the cerebrum is damaged, than the internal senses – imagination, memory, thought – suffer; the very will is weakened, and the power of determination is blunted. This is not the case if the injury is to the back part of the cerebrum” (1741)^9^.
**Jiri (Georg) Prochaska** (1749-1820) Czech anatomist and histologist	Prochaska, regarding the so-called higher functions, stated: ‘…since the brain…is composed of many parts, variously featured, it is probable, that nature…has dedicated these parts to various uses…the various ‘cogitating (intellectual) parts’ [faculties] (*diversae cogitationis partes*) seem to require different portions of the cerebrum…by no means improbable, that each division of the intellect [higher nervous functions] has its allotted organs, so that there is one for the perceptions, another for the understanding, probably others for the will, and imagination, and memory…”^11^^,^ 12.
**Franz Joseph Gall** (1758-1828) German physician and anatomist	Gall, in the initial outline of his theory, wrote: ‘…the faculties and propensities of man have their seat in the brain…the faculties among themselves, and the propensities among themselves, are essentially distinct and independent - they ought, consequently, to have their seat in parts of the brain distinct and independent of each other” (1798)^13^. He proposed that the brain possess 27 basic functions or ‘mental faculties’…associated to ‘organs’ [hypothetic cortical modules], distinguished by their functional strength (power), the real representative of the faculties, topographically localized on the brain surface [cerebral cortex]. The intellectual faculties (e.g., memory [words (verbal), things, facts (events), places (spatial), persons, numbers], senses [order, language (speech), places (spatial orientation), relation of numbers, persons) were localized in relation to the frontal bone, i.e., in the anterior [frontal] region (1810-1819)^14^.

## Theoretical-based view of localization: Swedenborg, Prochaska, and Gall

These authors considered the existence of diverse functions related to distinct cerebral seats where they were supposedly localized, and their theories shared many similarities. However, evidence supporting their presumptive localizations was not robust.

According to Emanuel Swedenborg, different functions must be represented in different anatomical seats at the level of the cerebral cortex. His biological manuscripts (1738-1744) remained unknown for more than one century, and despite finally being published (1882-1887), their impact was delayed.^9^^,^ 10 Jiri (Georg) Prochaska then considered that the various ‘cogitating parts’ (faculties) required different portions of the cerebrum, but without mentioning a specific localization for them (1779-1784).^11^^,^ 12 Finally, Franz Joseph Gall (with collaboration of Johann Gaspar Spurzheim), recognized distinct functions related to cerebral ‘organs’, perceived via reliefs of the head (skull), according to his peculiar localization criteria.^13^^,^ 14 He assigned higher (cognitive) functions to the anterior part of the head (frontal ‘organs’, frontal bone) (1810-1819).^14^ Gall’s (and Spurzheim’s) organology, which initially influenced many authors (e.g., Bouillaud, Dax, and others), were discredited relatively quickly. However, his ideas were recognized by many as the main forerunners of the concept of localizationism.^2^^,^ 10

## Brain lesion-based view of localization: from Wepfer to Dax

These were clinical and clinicopathological studies about brain lesions, represented mainly by cases of apoplexy (hemorrhagic, ischemic), head trauma, and expansive processes. The localization, when mentioned, was only touch on. The higher, better recognized function was related to the language domain^15^ (Box 3).

**Box 3:** Brain lesion-based view of localization, findings and excerpts from the authors.

Author	Function and localization
**Johannes Jakob Wepfer** (1620-1695) Swiss physician and pharmacologist	Wepfer explained that clinical manifestations in apoplexy should be seen as resultant from lesion of the brain parenchyma (*causa medullosa*), abandoning the ‘ventricular theory’ (1658) 17 ^,^ 18. He described numerous cases, at least 13 with speech disorders (aphasic type), with right hemiplegia, published posthumously by his nephews (*nepotum*) Bernhard and Georg Michael Wepfer (1727)^17^^,^ 19..Illustrative post-apoplexia [vascular] case (n^o.^ CLXVII) (1692): ‘…after headache…acute right hemiplegia, and loss of speech…partial recovery of movements… speech limitation, uttering only the word ‘Yes’ (*Ja*), able to see and recognize, unable to read…”, he identified as ‘right hemiplegia with speech loss’ (*hemiplegia lateris dextri cum loquelae abolitione*)^17^^,^ 19.Illustrative post-traumatic case (n^o.^ XVII) (1680): ‘...left bregmatic [frontoparietal] trauma…penetrating …causing a lesion of the brain substance…later almost speechless, uttering not more than affirmative or negative monosyllables, the right arm being paralyzed…”^17^^,^ 19.
**Antonio Maria Valsalva** (1666-1723) Italian physician	Valsalva stated: ‘...those afflicted by apoplexy, where one half of the body is paralyzed, the lesion occurred in the brain on the other side...”, regarding a patient who suffered an apoplexy. He affirmed that this ‘rule’ was confirmed by many autopsied cases (1707)^20^, published posthumously by Morgagni (1761). Many presented single-side paralysis (mostly right, and less left), with lesion contralateral to the hemiplegia, some with speech disorders [a few with possible aphasia] (mostly with right-side paralysis, and a few with left); on autopsy: intraventricular blood or serosity, serosity [ischemia] or blood [hemorrhage] over the hemisphere, lesion of the striatum, and/or thalamus^21^^,^ 22. Illustrative case VZ (1700): ‘…motor weakness on the right side, next loss of speech… right side became motionless [right hemiplegia]…aphonia improved, but speech was unintelligible and the words uttered with effort and a low voice”; on autopsy: ‘…fluid on the hemispheres, more on the right side (serous apoplexy)” [ischemic apoplexy] 21 ^,^ 22.
**François Pourfour du Petit** (1664-1741) French physician and anatomist	Pourfour du Petit examined five soldiers with traumatic head injuries, and limb paralysis opposite to the brain lesion, and later autopsied. He also found, on dissection, crossing fibers at the level of the pyramidal bodies (*corps pyramidal*) [pyramids] [medulla oblongata] as the anatomical substrate for this finding^24^. One of the cases also presented a speech disorder: ‘…on examination presented right hemiplegia… could not pronounce (utter) anything other than ‘No’ (…*mais il ne pouvoit prononcer autre chose que Non*)…judgement was always very sound during the illness…”; on autopsy: ‘…in the left side, the entire anterior protuberance [frontal lobe] including the…‘fluted bodies’ (*corps cannelez*) [corpus striatum] [and surrounding structures], was completely dissolved and reduced to a substance similar to wine lees…” (1710)^24^.
**Giovanni Battista Morgagni** (1682-1771) Italian physician	Morgagni grouped his own cases together with those of Valsalva and friends, all with hemiplegia, mostly right (some left) and contralateral brain lesions; some presented speech disturbances [a few could be identified as possible aphasia] (most with right-side paralysis, and a few with left-side); on autopsy: intraventricular blood or serosity, serosity [ischemia] or blood [hemorrhage] over the hemisphere, lesion of the striatum, and/or thalamus (1761)^21^^,^ 22. He affirmed: ‘…the dogma of Valsalva is confirmed…where a paralysis was observed on the left, a defect in the brain was found on the right…”^22^^,^ 23.Illustrative case: ‘…sudden right hemiplegia…scarce and babbling speech, replied with gestures when questioned, internal reasoning and understanding preserved…”; on autopsy: ‘…gelatinous material on the left side of the brain, two places eroded…the striated body, affected by the erosion…separated from the rest of the brain…”^21^^,^ 22.
**Jean-Baptiste Bouillaud** (1796-1881) French physician	Bouillaud initially observed cases presenting loss of speech, preserved understanding, and expression through writing or gestures, with lesion of the anterior lobe of the brain [without mentioning the side] on autopsy. He then presented cases with speech disorders, lesion of the anterior lobe (left [2] and right [1]) [no limb paralysis mentioned]. He also collected cases from other authors (Lallemand and Rostan), including cases with lesions to left (mostly), right or both anterior lobes (1825)^19,25,26,27^. Proposed the existence of a center related to speech function localized in the anterior lobes (*lobules antériores*) [frontal lobes] of the brain, the ‘legislator organ of speech” (*organe législateur de la parole*) (1825)^25^^,^ 26..
**Marc Dax** (1770-1837) French physician	Dax presented [allegedly] a report (1836), published posthumously (1865), on three patients with loss of ‘memory for words’ (one maintaining the ‘memory for objects”), one after a left parietal head injury, another after an apoplexy, with a lesion on the left brain surface on autopsy, and a third with ‘forgetfulness of words’, without anatomical definition. He believed that this pattern could be a ‘general rule’. However, he felt that these cases were insufficient and also respected the functional symmetry of the hemispheres doctrine (Bichat’s). After 3 other similar cases, and collecting about 80 more, without exception to the ‘rule’, he concluded: ‘…I believe…that not all diseases of the left hemisphere must impair the ‘memory for words’ [aphasia], but that, when this kind of memory is impaired by an illness of the brain, it is necessary to look for the cause of the disorder in the left hemisphere…”. He inquired: ‘...how is it that changes in the left cerebral hemisphere are followed by forgetfulness of words, while those in the right hemisphere are not?” (...*d’où vient que les altérations de l’hémisphère cérébral gauche sont suivies de l’oubli des mots, à l’exclusion de celles de l’hémisphère droit?*) ([1836]1865)^29^.

Seen as a pioneer of clinicopathological studies,^16^ Johannes Jakob Wepfer, described numerous cases, most published posthumously (1727).^17^^,^ 18 Vascular apoplexies had presumed brain lesion contralateral to the side of paralysis (as previously proposed by Hippocrates), and the side of a lesion causing a speech disorder could be inferred by the hemiplegia, or by the side of the head injury of traumatic cases.^19^ However, the lack of pathological verification of these cases precluded any description of the localization of the brain lesion.

Wepfer, seemingly, was not explicitly in search of higher function localization, and apparently, did not understand the relationship between the language impairment and left hemisphere lesion. Indeed, he described, more than once, the presence of ‘right hemiplegia with speech loss’, without further comments.^19^ The same can be stated about most subsequent authors who described comparable cases, but without clearly mentioning the value of such findings, until the time of Bouillaud, Dax, and mainly of Broca.

Another outstanding author, Antonio Maria Valsalva, proposed the occurrence of hemiplegia resulting from contralateral brain lesion, affirming that this ‘rule’ was confirmed by many autopsied cases (1707), which were collected and later published by Morgagni (1761).^20^^-^ 22 Shortly after, Domenico Mistichelli demonstrated anatomically crossing fibers, akin to a woman’s tress (*treccia di donna*), at the level of the medulla oblongata (1709), explaining Valsalva’s rule’.^23^ The disciple and follower of Valsalva, Giovanni Battista Morgagni, presented similar cases and confirmed Valsalva’s observation of paralysis contralateral to a brain lesion (1761).^22^^,^ 23

Apparently, Morgagni and Valsalva were not especially interested in disorders of higher functions, making no further comments on the speech problems of their cases, focusing mainly on the hemiplegia opposite to a brain lesion.

An author that also confirmed this finding was François Pourfour du Petit, who examined soldiers with head wounds and opposite side paralysis, eventually followed by pathological examination. He complemented and confirmed his observations experimentally in dogs (1710).^24^ He also found, on dissection, crossing fibers at the level of the medulla oblongata, mirroring Mistichelli’s observation.^24^ One of his cases, besides right hemiplegia, presented an aphasic manifestation and a left-side cortical-subcortical lesion.^24^ However, he made no specific comment about this differentiated finding, as his main objective was to explain the contralateral paralysis after head trauma.

Apparently, Pourfour du Petit’s case was the first clear description of this clinicopathological picture - right hemiplegia with aphasic symptom and contralateral brain lesion. However, the repercussion of his finding was limited.

An admirer of Gall’s work, Jean-Baptiste Bouillaud proposed the existence of a center related to speech functions localized in the frontal lobes (1825).^25^^,^ 26 He failed to mention a possible hemispheric asymmetry, despite reporting cases with predominant left-side lesions (over right) mostly of the frontal lobe, when speech disorders were present.^27^ Such misinterpretation might be explained by his strict respect for the doctrine of functional symmetry of the hemispheres, a concept dominant at the time, advocated by François-Xavier Bichat.^28^

Finally, Marc Dax reported three cases that presented a loss of the ‘memory for words’ (aphasia), with lesion of the left side of the brain. He tentatively identified a pattern that could be a ‘general rule’. However, the small number of cases and Bichat’s theory of functional symmetry of the hemispheres, limited his hypothesis. Later, a fair number of additional cases, without exception to this ‘rule’, confirmed his idea. He concluded that the impairment of the ‘memory for words’ should be attributed to a lesion of the left hemisphere ([1836]1865).^29^

His conclusion and query about the hemispheric functional difference suggest that he considered the left hemisphere as specific for language disorders, a hemispheric asymmetry.

Thus, the work Marc Dax presented ahead (1836) [allegedly] of Broca, marked the beginning of the awareness of ‘functional asymmetry’ of the cerebral hemispheres related to a higher function (here, the cognitive domain of language). This view was reinforced by Gustave Dax, who entered a dispute with Broca in defense of his father’s priority of this proposition.^8^^,^ 28 ^,^ 29

In conclusion, the period spanning about two centuries between Willis and Broca was marked by numerous authors who published works important for creating an atmosphere that permitted demonstration of the functional asymmetry of the hemispheres (Dax and Broca) and revealed the brain localization of a higher function in the left hemisphere as the language domain (Broca).

In the ensuing few decades, other localizations for further aspects of language were revealed by the likes of Wernicke and Lichtheim. Later, similar findings related to other higher functions (cognitive domains) appeared in publications by Liepmann, Wilbrand, Lissauer, Ribot, Korsakoff, among others.^30^
